# Influence of Novel SrTiO_3_/MnO_2_ Hybrid Nanoparticles on Poly(methyl methacrylate) Thermal and Mechanical Behavior

**DOI:** 10.3390/polym16020278

**Published:** 2024-01-19

**Authors:** Houda Taher Elhmali, Ivana Stajcic, Aleksandar Stajcic, Ivan Pesic, Marija Jovanovic, Milos Petrovic, Vesna Radojevic

**Affiliations:** 1 Faculty of Technology and Metallurgy, University of Belgrade, 11000 Belgrade, Serbia; alhmalyhdy384@gmail.com (H.T.E.); marija.jovanovic@tmf.bg.ac.rs (M.J.); mpetrovic@tmf.bg.ac.rs (M.P.); vesnar@tmf.bg.ac.rs (V.R.); 2 Department of Physical Chemistry, “Vinča” Institute of Nuclear Sciences—National Institute of the Republic of Serbia, University of Belgrade, Mike Petrovića Alasa 12-14, P.O. Box 522, 11001 Belgrade, Serbia; 3 Center for Microelectronic Technologies, Institute of Chemistry, Technology and Metallurgy—National Institute of the Republic of Serbia, University of Belgrade, 11000 Belgrade, Serbia; stajcic@nanosys.ihtm.bg.ac.rs (A.S.); ivan.pesic@ihtm.bg.ac.rs (I.P.)

**Keywords:** nanoparticles, SrTiO_3_/MnO_2_, PMMA composite, mechanical properties

## Abstract

While dental poly methyl methacrylate(PMMA) possesses distinctive qualities such as ease of fabrication, cost-effectiveness, and favorable physical and mechanical properties, these attributes alone are inadequate to impart the necessary impact strength and hardness. Consequently, pure PMMA is less suitable for dental applications. This research focused on the incorporation of Strontium titanate (SrTiO_3_-STO) and hybrid filler STO/Manganese oxide (MnO_2_) to improve impact resistance and hardness. The potential of STO in reinforcing PMMA is poorly investigated, while hybrid filler STO/MnO_2_ has not been presented yet. Differential scanning calorimetry is conducted in order to investigate the agglomeration influence on the PMMA glass transition temperature (T_g_), as well as the leaching of residual monomer and volatile additives that could pose a threat to human health. It has been determined that agglomeration with 1 wt% loading had no influence on T_g_, while the first scan revealed differences in evaporation of small molecules, in favor of composite PMMA-STO/MnO_2_, which showed the trapping potential of volatiles. Investigations of mechanical properties have revealed the significant influence of hybrid STO/MnO_2_ filler on microhardness and total absorbed impact energy, which were increased by 89.9% and 145.4%, respectively. Results presented in this study revealed the reinforcing potential of hybrid nanoparticles that could find application in other polymers as well.

## 1. Introduction

**Scheme 1:**
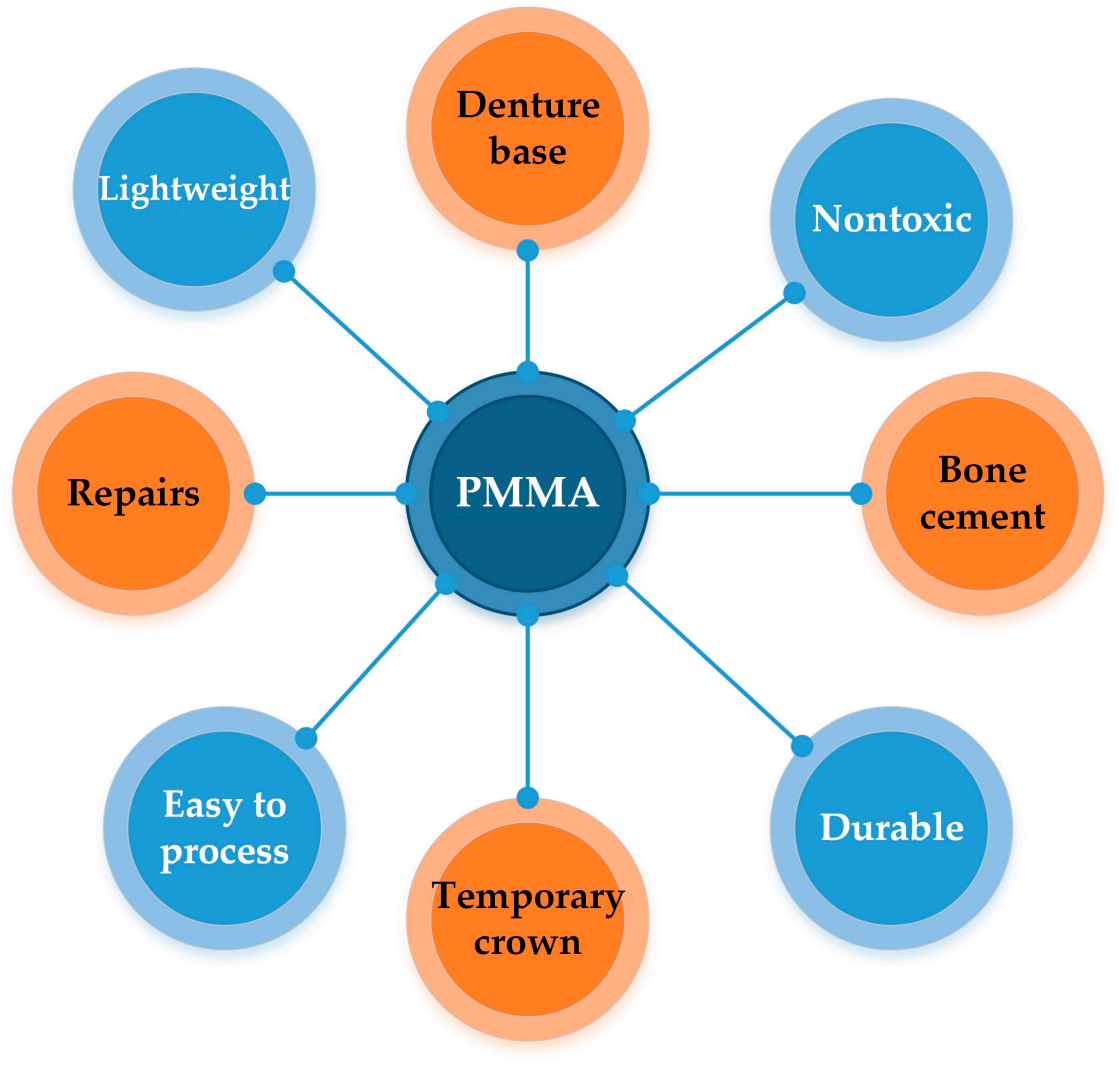
Advantages and applications of PMMA in dentistry.

Various polymers have widespread use in the field of dentistry, from bone cement materials or repairs to drug delivery [1,2,3]. Among these, poly methyl methacrylate (PMMA) stands out as a versatile polymer extensively utilized for crafting orthodontic retainers, dentures, and repairs, as well as artificial teeth [4,5,6]. Scheme 1 illustrates an array of advantages and applications of PMMA. Years of research and practical application have established that PMMA provides durability, nontoxicity, ease of processing, and hence, economic viability, as well as high biocompatibility [7].

However, along with the noted advantages, there are important drawbacks in mechanical properties that hinder widespread PMMA application [8,9], leading to studies on the improvement of mechanical strength, toughness, impact resistance, and hardness [10]. One of the approaches to enhance the mechanical performance of PMMA is by introducing fillers that can tune both, mechanical and functional properties. Numerous studies have presented improvements in targeted PMMA properties materials by incorporating fibers or nanoparticles [11,12,13,14,15,16,17]. Among the versatile selection of nanoparticles, silica (SiO_2_), alumina (Al_2_O_3_), titania (TiO_2_), and zirconia (ZrO_2_) are the most investigated as reinforcements for PMMA, due to their mechanical and antibacterial potential [17,18,19,20,21,22]. These particles are also known for their ability to form various surface architectures at microscopic and nanoscopic levels, which further enables control of various functional properties [23]. Vojdani et al. improved hardness by 13% with the addition of 5 wt% of alumina to PMMA [24]. Hata et al. reported a more than 100% increase in hardness with 23% of nanoporous silica [25]. On the other side, Alhotan et al. achieved only a 10% hardness increase with 7% of nano-zirconia [26]. Furthermore, Gad et al. discovered that, although ZrO_2_ increases flexural properties, it also decreases the impact strength of PMMA [27].

In order to enhance mechanical properties and overcome challenges involving the enhancement of one property at the expense of another, research has been focused on the hybrid concept i.e., combinations of different particles, nanotubes, or fibers, with the aim of achieving a synergistic effect between the reinforcements. Recently, Naznin et al. increased the tensile strength of PMMA by 329% by incorporating graphene oxide and mesoporous micro-silica (SiO_2_) [28]. Chen et al. incorporated nanoparticles of TiO_2_ and micro poly(ether ether ketone) (PEEK) in order to increase the bending strength and flexural modulus of PMMA. With 1 wt% of TiO_2_ and PEEK, both flexural strength and modulus increased by 10% compared to control PMMA [29]. Alqahtani et al. reported that with a 10% load of Zirconia-Yttria (ZrO_2_-Y_2_O_3_) hardness of PMMA was increased 4.5 times, while flexural strength showed an increase of 7 times [30]. The same group incorporated hexagonal boron nitride (h-BN) in PMMA, varying concentration and particle size [31]. They reported a 2.7 times increase in hardness with 5 wt% of h-BN loading. By using hybrid reinforcement Al_2_O_3_/TiO_2_ nanoparticles, Nabhan et al. increased the fracture toughness of PMMA by 10% with only 1.6 wt% of the filler [32]. According to Wu et al., with 9% of nano hydroxyapatite and 12.5% nano alumina, a 40% increase in fracture toughness could be achieved [33]. Obviously, applying multiple reinforcements within the same polymer matrix brings more benefits than using individual fillers. Additionally, hybrid particles can enable the enhancement of biocompatibility and bioactivity, as shown by Beketova et al. in the investigation of the ZrO_2_-Y_2_O_3_ system [34].

Numerous ongoing studies are focused on processing PMMA-based nanocomposites, where there are still many challenges to improving mechanical properties without disrupting biocompatibility or cost-effectiveness. A perovskite-structured oxide called strontium titanate (SrTiO_3_-STO) is known for its excellent piezoelectric, dielectric, and thermoelectric properties, as well as photocatalytic activity [35,36,37]. However, it has also been recognized in medicine, due to biocompatibility and bioactivity, as an osteogenesis promoter; furthermore, STO exhibits antibacterial activity, which makes it a valuable material for implants [38,39,40]. Si et al. showed that STO promotes the improvement of tribological properties, as well as the antibacterial activity when applied on titanium-based implants [41]. The potential of nanoparticle STO application in dental materials has still been limited to implant coatings, although it could offer advantages in other fields of medicine, as it has exceptional mechanical properties, such as high Young’s modulus, compressive strength, and hardness [36,42].

Another oxide that could be valuable for tuning PMMA properties is manganese oxide (MnO_2_), due to its nontoxicity, antibacterial activity, resistance to acids, and low cost [43,44,45,46]. Although MnO_2_ has mostly been researched for its catalytic activity, its use in medicine and environmental protection was recently emphasized as well [47,48]. There are very few studies on the mechanical benefits of the incorporation of MnO_2_ in polymer matrices. However, Balguri et al. recently reported that with only 0.1 wt% of MnO_2_, the impact strength of epoxy was improved by 35% [49]. Zhao et al. incorporated MnO_2_ in polyvinylidene fluoride (PVDF) in order to enhance the piezoelectric and mechanical performance of composites [50]. With 1.5 wt% loading of MnO_2_, Young’s modulus was increased by 75%, showing the reinforcing potential of this oxide.

While there is obvious potential for STO and MnO_2_ nanoparticle-reinforced PMMA, there is a lack of research investigating the influence of these ceramic materials on the mechanical properties of PMMA, with a special emphasis on impact resistance and hardness, which are crucial for dental materials. This research focuses on the mechanical performance of PMMA reinforced with STO and hybrid STO/MnO_2_ nanoparticles. Obtained composites showed significant improvement in Young’s modulus of elasticity, hardness, and impact resistance.

## 2. Materials and Methods

### 2.1. Materials

SrTiO_3_, nanopowder and MnO_2_ were purchased from Sigma–Aldrich. PMMA was purchased from AKRILAT, Serbia. Commercial dental acrylic resin is made of two components system, liquid and powder. The liquid part contains methyl metacrylate (MMA)—monomer and inhibitor, while the powder contains PMMA, initiator, plasticizer, and pigments. Upon mixture of the two parts, the initiator neutralizes the effect of the inhibitor allowing the polymerization reaction to start.

### 2.2. Preparation of Samples

SrTiO_3_/MnO_2_ (STO/MnO_2_) nanoparticles were prepared by milling for 30 min and heating for 2 h at 1000 °C, with a 5 °C/min heating rate, in N_2_ atmosphere. The molar ratio SrTiO_3_:MnO_2_ was 5:1.

Composites were prepared in three steps:

- Particles were placed in a liquid part containing MMA, and mixed on a magnetic stirrer, followed by ultrasonic homogenization.
- The liquid part with STO/MnO_2_ nanoparticles was mixed with powder in a volumetric ratio of 35/100, in accordance with the manufacturer’s instructions.
- The mixture was poured into a mold and left for 24 h at room temperature.

The overall concentration of nanoparticles was 1 wt%. Samples were labeled as follows: PMMA, PMMA-STO, and PMMA-STO/MnO_2_.

### 2.3. Characterization of Samples

For morphological analysis of samples, field emission scanning electron microscopy (FESEM) was utilized with Tescan Mira 3 instruments (Brno, Czech Republic), where gold was sputtered prior to imaging. Particle size was determined by analysis of three FESEM images using the software *Image-Pro Plus 6.0* (Rockville, MD, USA). Structural analysis of STO/MnO_2_ nanoparticles was performed using an X-ray diffractometer (XRD) Ultima IV, Rigaku (Tokyo, Japan). The measurement was performed using Cu Kα radiation, with the following measurement parameters: 2θ angle range from 20° to 80°, speed 10° min^−1^, step scan 0.02°. For the structural analysis of PMMA and composites, Fourier transform infrared spectroscopy (FTIR) was performed using a Thermo Scientific Nicolet iS10 spectrometer (Hartmann & Braun, MB-series, Bockenheim, Germany), in the range from 4000 to 500 cm^−1^, with a resolution of 4 cm^−1^. Differential scanning calorimetry (DSC) was performed on Shimadzu DSC-60Plus (Kyoto, Japan). The sample weight was 6 ± 0.5 mg. Initially, the samples underwent heating from 24 °C to 160 °C, with a heating rate of 10 °C/min in the presence of a nitrogen gas flow (50 mL/min). Subsequently, the second heating cycle was conducted under identical conditions. The data from the second heating cycle were used for the determination of T_g_. Tensile test of pure PMMA and composites was obtained by texture analyzer Shimadzu EZ Test LX, in accordance with ISO 527-2 standard (Kyoto, Japan) for plastics. The device was equipped with a 500 N load cell, applied strain rate was 10 mm/min. Cross-section area and gauge length were measured before each test. *Trapezium 1.5.2* software (Duisburg, Germany) by Shimadzu was used for the calculation of modulus of elasticity and tensile strength. All of the measurements were performed at room temperature. Microindentation testing was performed on the same device, with the following parameters set: 500 N load cell, a spherical indenter of 4 mm diameter, indentation rate of 0.25 N/s. The maximum load of 5 N was kept for 20 s, after which was decreased at the same rate of 0.25 N/s. The output results were force, time, and relative position of the indenter. Measurements were performed on three points for each sample, to reduce the influence of possible inhomogeneity. High-speed puncture impact testing machine HYDROSHOT HITS-P10, Shimadzu (Kyoto, Japan), was used for the investigation of nanoparticle influence on the impact resistance of PMMA. Absorbed energy (total absorbed energy-E_tot_ and energy at maximum load-E_fmax_) values were calculated automatically from the load-time diagram. The diameter of the striker was 12.7 mm, with a hemispherical head; the set impact force was 10 kN, and impact velocity and depth were 0.15 m/s and 1 mm, respectively. For impact testing, samples had dimensions 6 × 6 × 3 cm^3^. Each measurement was performed on three samples, with the average values noted in the results.

## 3. Results and Discussion

### 3.1. FESEM of STO/MnO_2_ Nanoparticles

**Figure 1:**
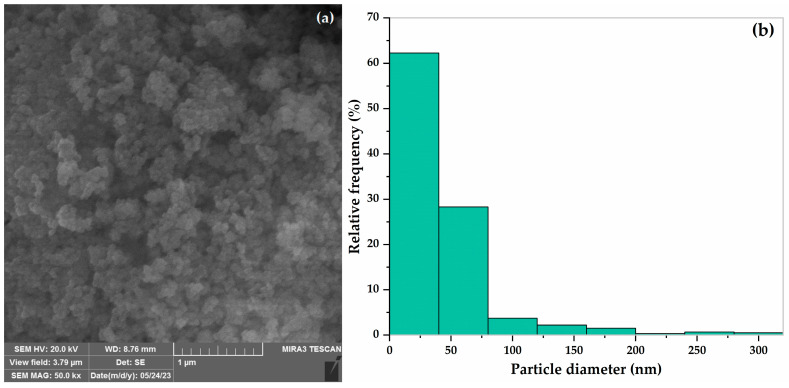
(**a**) FESEM of STO/MnO_2_ (**b**) Particle size distribution.

Figure 1a shows an FESEM image of prepared STO/MnO_2_ particles. As can be seen, spherical-shaped nanoparticles formed aggregates due to electrostatic forces, coming from STO that is dominant in our sample [51]. Image analysis showed that the average particle diameter was around 48 nm, with a modal value of approximately 35 nm. Distribution of particle size (Figure 1b) revealed that more than 60% of the particles had diameters below 50 nm and 93% were below 100 nm, which suggests a successful synthesis of nanoparticles that could contribute to the mechanical reinforcement of PMMA.

### 3.2. XRD of STO/MnO_2_ Nanoparticles

**Figure 2:**
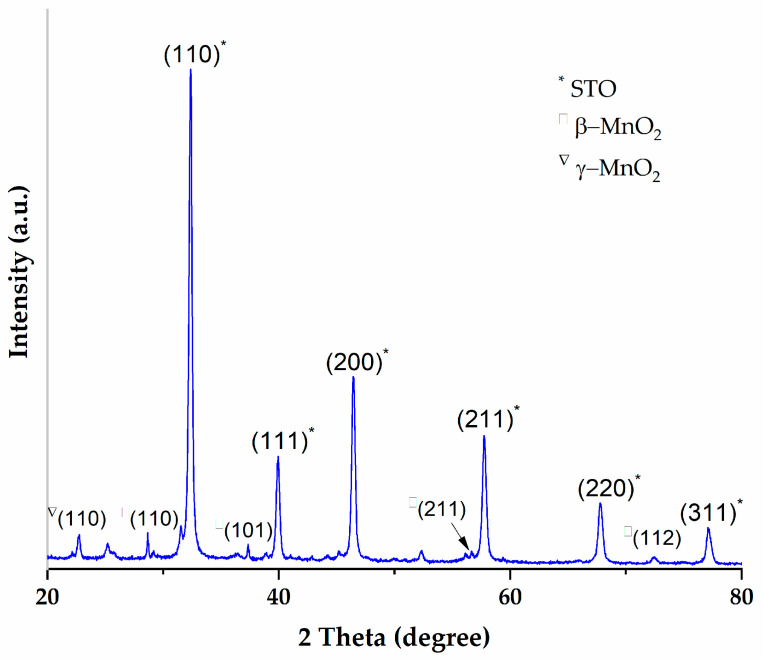
XRD of SrTiO_3_/MnO_2_ particles.

Figure 2 shows the diffraction pattern of STO/MnO_2_ particles. The strongest peaks observed in the STO/MnO_2_ are detected at angular positions of 32.4, 40.0, 46.3, 57.8, 67.8, and 77.0°. These peaks are attributed to the crystallographic planes of STO possessing cubic symmetry, specifically corresponding to (110), (111), (200), (211), (220), and (311) orientations [35,52]. Diffraction originating from MnO_2_ also shows four peaks at 28.8, 37.2°, 56.7° and 72.4°attributed to the (110), (101), (211) and (112) planes of *β*-MnO_2_ polycrystalline nanoparticles consisting of pure single tetragonal phase system [53,54]. The peak at 22.7° probably belongs to orthorhombic γ-MnO_2_, which also exhibits peaks at 37.2°, 56.2, and 67.8° that overlap with *β*-MnO_2_ and STO [55,56,57]. Residual SrCO_3_ in commercial STO was detected at 25.1°, corresponding to the (111) plane, which remained present even after the heat treatment at 1000 °C [58].

Results obtained by XRD analysis indicate that synthesized nanoparticles were STO/MnO_2_ composites, with crystal lattices intact. In this manner, properties of the separate compound should be retained and the possibility of synergistic effect enhanced. Furthermore, two phases of MnO_2_ were identified, *β*-MnO_2_ and γ-MnO_2_, as a consequence of high-temperature treatment.

### 3.3. FESEM of PMMA and Composites

**Figure 3:**
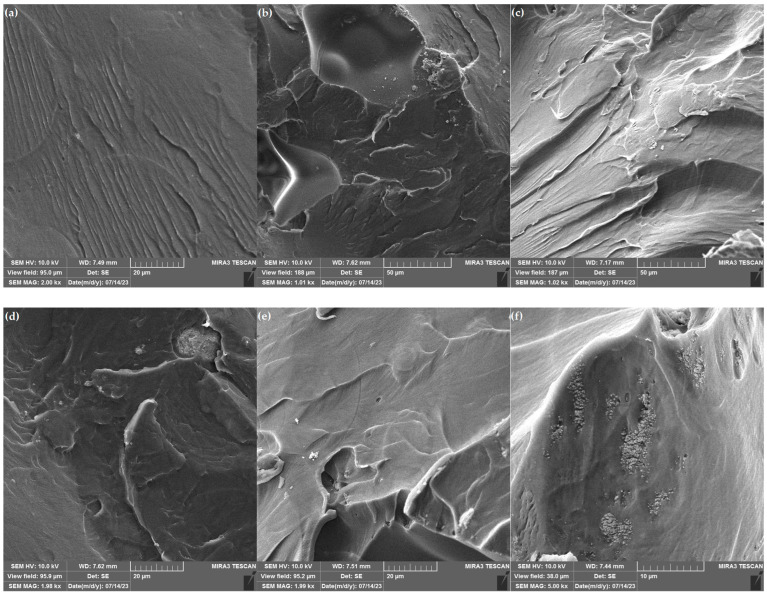
FESEM images of (**a**) PMMA (2000× magnification); (**b**) PMMA-STO (1000× magnification); (**c**) PMMA-STO/MnO_2_ (1000× magnification); (**d**) PMMA-STO (2000× magnification); (**e**) PMMA-STO/MnO_2_ (2000× magnification); (**f**) PMMA-STO/MnO_2_ (5000× magnification).

Morphological analysis of the fracture site was performed on FESEM images presented in Figure 3. Particle-reinforcing effects in composites play a crucial role in determining their mechanical properties, especially at the fracture site [59,60]. During polymerization, various inter-constituent changes occur, such as twisting, bending, and flexing, which influence particle-polymer interfacial surface and consequently, mechanical properties, such as toughness and elastic behavior [61]. The following crucial toughening mechanisms can be identified in FESEM images [59]:

- Crack Bridging: Particles can bridge microcracks that form within the composite matrix. As a crack attempts to propagate, it encounters bridges, which resist further crack growth. The bridging mechanism increases the energy required for crack propagation enhancing the composite’s fracture resistance.
- Crack Deflection: Particles can cause cracks to change direction when they encounter the particle-matrix interface. This deflection reduces the effective crack length, increasing the composite’s resistance to fracture and improving its toughness.
- Crack tip pinning: As the crack propagates and encounters a pinning point, it experiences local resistance. This resistance arises due to the additional energy required to deform the material or overcome the obstacles presented by the pinning point. The energy required to deform the material around the pinning point, or to move the crack past the obstacle, is dissipated as heat. This energy dissipation contributes to the overall toughness of the material.

Comparison of PMMA morphology with composites clearly shows the reinforcing effect of both STO and STO/MnO_2_ particles. Pure PMMA surface (Figure 3a) shows a typical brittle fracture, with smooth fracture surface and river-like patterns, where plastic deformation plays a key role in fracture resistance [62]. On the other hand, both composites, PMMA-STO and PMMA-STO/MnO_2_ (Figure 3b,c), show different toughening mechanisms. The fracture surface of both PMMA-STO and PMMA-STO/MnO_2_ showed higher roughness compared to PMMA, which is directly connected to a reinforcement in the material. The inclusion of the filler in the matrix microstructure restricts dislocation movement during loading, thereby enhancing the strength and modulus of the composites [32]. Formation of aggregates was observed, which also served as a reinforcement-inducing crack-pinning mechanism [63]. Strong plastic deformation also reveals increased fracture resistance. The dominant toughening mechanism was crack-pinning in both composites, whereas pure STO particles formed large aggregates that participated in crack arrest through plastic void formation as well [64]. On the fracture surface of PMMA-STO/MnO_2_, small aggregates were formed, indicating better dispersion of hybrid nanoparticles in composite, presumably due to the formation of a larger interfacial surface [61].

By increasing the magnification from 1000× to 2000× (Figure 3d,e), an obvious difference in agglomeration was observed. While STO/MnO_2_ showed some uniformity in agglomerate size, pure STO showed a wide range of sizes, from around 1 µm to 20 µm. This could be the consequence of weaker van der Waals forces in hybrid nanoparticles compared to pure STO. However, in PMMA-STO/MnO_2_, a group of smaller agglomerates surrounded by a polymer matrix was found with 5000× magnification, which caused significant plastic deformation and void formation as a toughening mechanism. Based on FESEM analysis, it was expected that both STO and PMMA-STO/MnO_2_ nanoparticles would contribute to the reinforcement of the matrix material.

### 3.4. FTIR of PMMA and Composites

**Figure 4:**
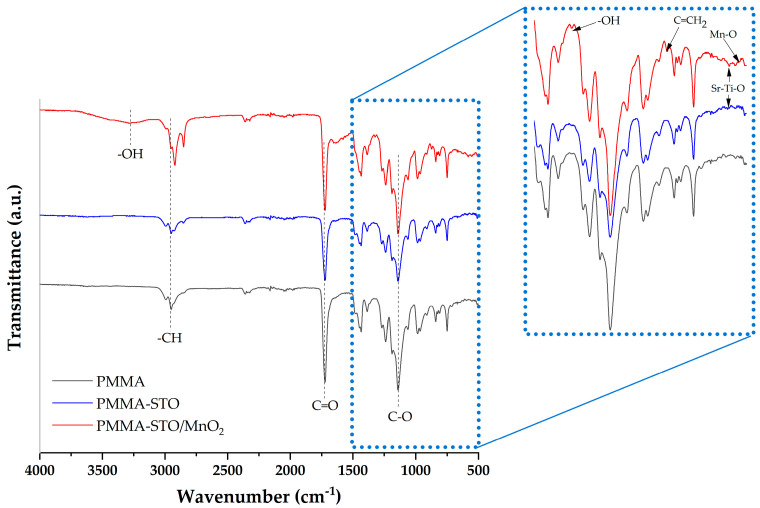
FTIR spectra of PMMA and composites with enlarged region 1500–500 cm^−1^.

In Figure 4, the infrared spectra of the pristine PMMA, PMMA-STO, and PMMA-STO/MnO_2_ are presented. In order to emphasize the differences between spectra, especially in the fingerprint region, region 1500–500 cm^−1^ was enlarged in Figure 4. The asymmetric stretching of C-H bonds, present in -CH₃ manifests itself at 2995 cm^−1^ in all spectra [65]. At 2925 and 2856 cm^−1^, asymmetric and symmetric C-H stretch in -CH_2_ was observed, respectively. Notably, the stretching vibration of the ester group’s C=O bond is prominently evident at approximately 1721 cm^−1^ [66]. The strong peak at 1430 cm^−1^ originates from the asymmetric bending vibrations of the C-CH₃. Two sets of doublet bands, one at 1267 and 1241 cm^−1^ and the other at 1143 and 1183 cm^−1^ can be attributed to the C-O stretching vibrations within the ester groups [67,68]. Out-of-plane -CH bending is present in the region from 900 to 720 cm^−1^ [62,69]. In the spectrum of PMMA-STO/MnO_2_, a minor band at 875 cm^−1^ could be associated with the bending of CH from C=CH_2_ in residual monomer methyl methacrylate, indicating that agglomerates act as interceptors for monomer during polymerization reaction. Furthermore, peaks visible at 3277 cm^−1^ originate from stretching vibrations of hydroxyl groups related to the STO/MnO_2_ nanoparticles [55]. Bands at 1637 cm^−1^ and 1320 cm^−1^ come from -OH bending. The most prominent peaks in the FTIR spectrum of STO are related to the stretching vibrations of Ti-O bonds. These peaks are typically observed in the range of 500–800 cm^−1^. Sr-Ti-O stretching in STO appeared at 580 cm^−1^ in both composites as a weak band, which is expected due to a low particle concentration [70,71]. Vibrations from Mn-O are visible at 536 cm^−1^ [72].

FTIR analysis revealed that hybrid STO/MnO_2_ nanoparticles had a minor influence on influence on polymerization of MMA. The identification of nanoparticles at randomly chosen sites indicates that some level of uniformity was achieved, without the chemical modification of particles.

### 3.5. Differential Scanning Calorimetry (DSC)

**Figure 5:**
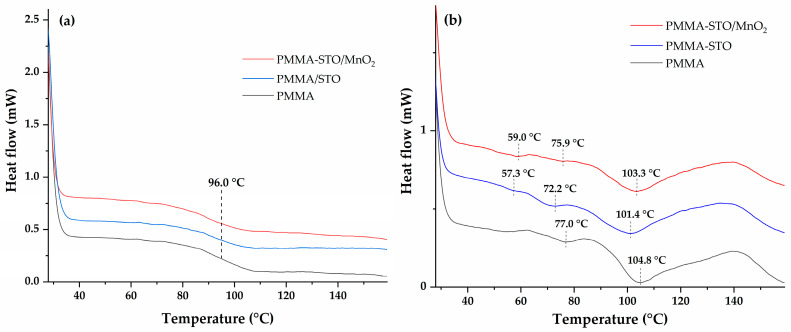
DSC analysis of PMMA and composites: (**a**) second scan; (**b**) first scan.

Particle agglomeration could lead to deterioration of mechanical properties, acting as stress concentration points that lead to a crack formation. Furthermore, large particles/agglomerates may act as plasticizers, increasing chain mobility and decreasing intermolecular forces that resist applied mechanical force. This is connected to thermal behavior, characterized by glass transition temperature (T_g_). In order to investigate whether agglomeration lead to a decrease in T_g_, DSC analysis was performed. Figure 5a shows that the formation of nanoparticle agglomerates had no significant influence on T_g_, which is around 96.0 °C in all samples. This finding is indicative and important for the mechanical performance of composites and is in agreement with the literature, where it has been proven that low particle concentrations do not lead to a decrease in composite T_g_.

The first scan presented in Figure 5b shows differences between samples during heating. In PMMA straight baseline was observed until 62.8 °C, where an onset for a weak endothermic peak at 77.0 °C was observed. Another endotherm was observed at 104.8 °C, with an onset at 84.0 °C. On the other side, PMMA-STO/MnO_2_ showed the first endotherm at 59.0 °C, followed by a weak one at 75.9 °C and a strong peak at 103.3 °C. PMMA-STO had a minor endotherm at 57.3 °C, stronger at 72.2 °C, and a major peak at 101.4 °C. The boiling point of MMA is 100 °C, which indicates that in pure PMMA, the monomer was trapped by the polymer chains more efficiently than in composites. Particle agglomerates enabled more mobility for MMA, leading to evaporation at temperatures close to the unhindered monomer. However, these temperature shifts are minor, which leads to the conclusion that there would not be a significant difference in monomer leaching [73]. Furthermore, although other weaker endotherms indicate that volatile additives are easier to release and leach in composites, in PMMA-STO/MnO_2_ there was a weaker endothermic peak compared to PMMA around 76 °C, which indicates that hybrid nanoparticles could more efficiently trap some of the smaller molecules. In light of the results based on thermal analysis, it can be assumed that STO/MnO_2_ agglomerates do not pose a problem from a medical point of view as well.

### 3.6. Mechanical Properties

Results obtained from tensile test, microindentation, and controlled energy impact test are presented in Table 1 and Figure 5. Modulus of elasticity increased by 39.6% in PMMA-STO and 44.0% in PMMA-STO/MnO_2_, compared to the pure PMMA. Tensile strength was approximately the same in PMMA-STO and PMMA-STO/MnO_2_, respectively, 29.4% and 29.9% higher than for PMMA. The interface that arises from the bonding between filler particles and polymer chains leads to enhanced mechanical properties in the modified composites compared to the pristine material [74]. Specifically, when STO and STO/MnO_2_ nanoparticles were employed to reinforce PMMA, a robust interface ensured that the applied load was transmitted to the resilient inorganic nanoparticles, subsequently distributing it across multiple polymer chains, safeguarding the matrix.

Microindentation measurements revealed that hardness was increased by 29.7% in PMMA-STO and by 89.9% in PMMA-STO/MnO_2_, which indicates that the small amounts of hybrid STO/MnO_2_ nanoparticles can significantly improve mechanical performance. Furthermore, reduced modulus increased by 45.0% in PMMA-STO and by 66.1% in PMMA-STO/MnO_2_. Compared to the studies that investigated the influence of alumina, silica, and zirconia with higher loadings, improvements observed in both composites show great reinforcing potential [26,27,28]. This phenomenon could be attributed to the considerably higher hardness of the particles in comparison to pure PMMA, thus contributing to an increased load-bearing capacity [32]. The difference in the results between PMMA-STO and PMMA-STO/MnO_2_ is a consequence of better STO/MnO_2_ dispersion in the matrix, which indicates that MnO_2_ contributed to better adhesion between the hybrid particles and the matrix.

The energy absorption process during impact can be divided into distinct phases, as follows [57]:

1. Initial Crack Formation Phase: In this first phase, energy is absorbed as the crack initially forms within the material. It encompasses both the elastic response of the material and any minimal plastic deformation. For fully cured epoxy resins, this phase is characterized by relatively low energy absorption. The maximum load (F_max_) is achieved at the conclusion of this phase, and the energy absorbed up to this point is denoted as E_fmax_.
2. Crack Propagation and Material Deterioration Phase: The second phase commences with the formation of the crack and extends until the material eventually fails or ruptures. During this phase, there is a notable degradation of mechanical properties. The total absorbed energy (E_tot_) encompasses all the energy absorbed from the start of the controlled energy impact test until the load drops below zero, marking the conclusion of the test.

These phases are associated with different types of failure, depending on the material’s propensity for plastic deformation:

- Brittle Failure: This type of failure is characteristic of materials like ceramics and rigid polymer structures, such as cross-linked polymers forming 3D covalently bonded networks. Brittle failure is characterized by minimal or no plastic deformation, rapid fracture propagation, and a low E_tot_ value.
- Brittle-Ductile Fracture: In this scenario, there is a limited degree of plastic deformation that occurs just before the material breaks. It represents an intermediate stage between brittle and ductile behaviors.
- Ductile-Brittle Failure: Materials exhibiting this type of failure have the capacity for plastic deformation and can absorb more energy during impact compared to brittle materials. They undergo some plastic deformation before ultimately fracturing.
- Ductile fracture: The fourth type of failure is characterized by substantial plastic deformations occurring before fracture. Materials that exhibit this behavior absorb a significant amount of energy during the impact, resulting in a high E_tot_ value.

**Figure 6:**
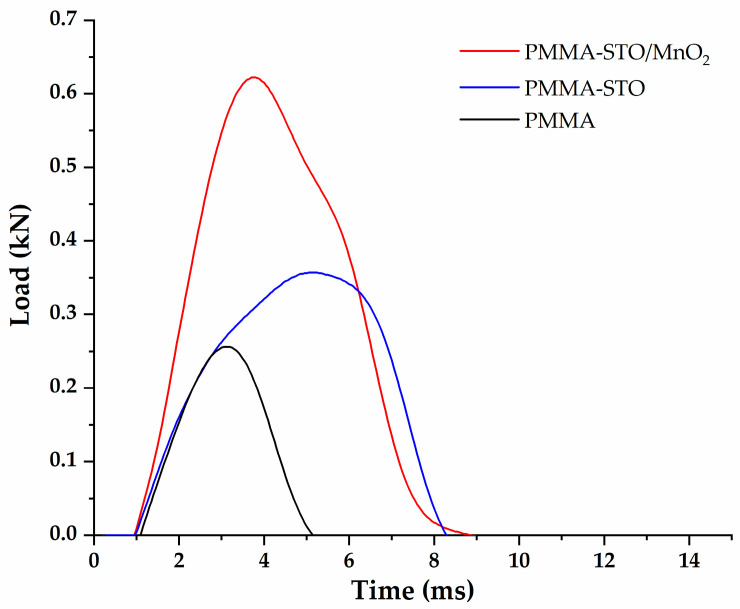
Load-time curves obtained from controlled energy impact test.

These distinctions in failure modes and energy absorption characteristics are crucial for understanding how different materials respond to impact loads and for assessing their suitability for various applications. Figure 6 shows failure types for PMMA, PMMA-STO, and PMMA-STO/MnO_2_. PMMA shows brittle failure, which is expected since it is considered a brittle thermoplastic polymer [75]. With the addition of STO, fracture changed to brittle-ductile, which is the consequence of energy dissipation during crack pinning and plastic void formation, observed on FESEM images. In PMMA-STO/MnO_2_, failure mode became ductile-brittle, indicating that STO/MnO_2_ have stronger interaction with the matrix, which was indicated in FESEM analysis, where smaller aggregates were observed than in PMMA-STO. However, STO particles led to an increase of 72.7% in E_tot_, which is a significant improvement in impact resistance. Compared to Balguri et al., where a similar amount of pure MnO_2_ was used, the increase of 145.4% in total absorbed impact energy achieved in this study shows the remarkable effect of synergy between STO and MnO_2_ nanoparticles [49].

In order to verify the statistically significant mechanical improvement, the Student’s *t*-test was performed for the results presented in Table 1 [76]. Arithmetic means of control series (PMMA) were compared with PMMA-STO and PMMA-STO/MnO_2_. High t- and low *p*-values presented in Table 2 illustrate high statistical significance (threshold: t > 1.96, *p* < 0.05) of an increase in all the mechanical properties.

The results obtained from the tensile test, microindentation and controlled energy impact test revealed that there is a high potential for using novel STO/MnO_2_ nanoparticles as mechanical reinforcement. Furthermore, these results were obtained without the chemical modification of nanoparticles, which is usually required for significant mechanical improvements [75]. Since both STO and MnO_2_ have been already investigated and proven for their biocompatibility and antibacterial activity, the main scope of our future research will be on varying nanoparticle concentration and investigating antibacterial activity.

## 4. Conclusions

In this research, two types of nanoparticulate fillers, STO and hybrid STO/MnO_2_, have been used as reinforcements for dental (PMMA), with the aim of improving its low impact resistance and hardness. Hybrid STO/MnO_2_ nanoparticles were obtained by sintering at 1000 °C and incorporating them in the liquid part of the two-component dental PMMA resin, with 1 wt% concentration. Structural analysis of hybrid nanoparticles revealed the existence of cubic STO, as well as *β*-MnO_2_ and γ-MnO_2_. The results of DSC analysis indicated that agglomeration of nanoparticles with a 1 wt% loading had no significant impact on T_g_. However, the first DSC scan revealed disparities in the evaporation of small molecules, with some favoring PMMA-STO/MnO_2_ composite that demonstrated a potential for trapping volatiles. Morphological characterization revealed synergy between STO and MnO_2_ in STO/MnO_2_ nanoparticles, leading to a better dispersion in the matrix, compared to STO. As a consequence of a better dispersion, a higher improvement in PMMA mechanical performance was achieved. In the PMMA-STO composite, microhardness was increased by 29.9%, modulus of elasticity by 39.6%, and total absorbed impact energy (E_tot_) by 72.7%, which showed that even with the formation of aggregates, STO represents a promising reinforcement for PMMA. Composite PMMA-STO/MnO_2_ has shown an increase in microhardness by 89.9%, and modulus of elasticity by 44.0%, while E_tot_ values rose by 145.4%. These results indicated that a low concentration of novel hybrid STO/MnO_2_ nanoparticles could lead to an outstanding mechanical performance by a PMMA-based material.

## Figures and Tables

**Table 1:** Mechanical properties of PMMA and composites.

	PMMA	PMMA-STO	PMMA-STO/MnO_2_
Modulus of elasticity, MPa	720.6 ± 22.13	1006.0 ± 15.37	1037.3 ± 24.63
Tensile strength, MPa	23.1 ± 1.22	29.9 ± 1.86	30.0 ± 1.41
Hardness, MPa	14.8 ± 0.43	19.2 ± 1.82	28.1 ± 2.23
E_tot_, J	1.1 ± 0.16	1.9 ± 0.14	2.7 ± 0.21

**Table 2:** Results of Student’s *t*-test.

	PMMA-STO *		PMMA-STO/MnO_2_^‡^	
	t-Value	*p*-Value	t-Value	*p*-Value
Modulus of elasticity	18.3465	<0.0001	16.5664	<0.0001
Tensile strength	5.2949	0.0061	6.4097	0.0030
Hardness	4.0752	0.0152	10.1433	0.0005
E_tot_	6.5175	0.0029	10.4970	0.0005

* Group 1: PMMA, Group 2: PMMA-STO; n (number of measurements) = 3. ^‡^ Group 1: PMMA, Group 2: PMMA-STO/MnO_2_; n (number of measurements) = 3.

## Data Availability

The data presented in this study are available on request from the corresponding author. The data are not publicly available due to privacy.
